# Intelligence, Correspondence, &c.

**Published:** 1835-04-01

**Authors:** 


### INTELLIGENCE, CORRESPONDENCE, &c.

### ST. GEORGE'S HOSPITAL—NEW SCHOOL OF ANATOMY,

*Kinnerton Street, Knightsbridge, immediately adjoining St. George's Hospital.*

This splendid Building, with its extensive public and private Dissecting Rooms, will be opened for the purpose of Anatomical Instruction, on the 1st of *October*, 1835. Every possible arrangement has been made for securing the health and comfort of the Student.

The Lectures on Anatomy will be delivered by Mr. Tatum, and Mr. Henry James Johnson; the Demonstrations by the latter Gentleman, and Mr. Henry Charles Johnson.

---

Guernsey, *March* 16*th*, 1835.

Gentlemen,

In reply to an anonymous communication in your last Number I shall merely observe, that "the numerical details" which "cast a shade of reality" over the cases treated by salt and water, in our Cholera Hospital, are derived from the Register kept by the House Surgeon, under the direction of the late Board of Health; which document was placed at my disposal, during the prevalence of the disease, by a member of the very Board referred to by your Correspondent.

I am, Gentlemen,

Your obedient Servant,

S. E. HOSKINS.

*To the Editors of the Med.-Chir. Review.*

---

We are glad to perceive that Dr. John Hamett, the talented author of the statistical and practical Work on Epidemic Cholera, has been honoured with the Fellowship of the Royal Society—a distinction conferred on him, we understand, without a single dissentient voice.

---

*In the Press,*

A Clinical Account of Fever, Gout, Rheumatism, &c. and various Diseases of the Chest. By Dr. Aldis, Cantab. Member of the Royal College of Physicians, London.

